# Supplementary material for: Effect of Group Housing of Preweaned Dairy Calves: Health and Fecal Commensal Antimicrobial Resistance Outcomes
Source: Antibiotics (Basel). 2023 Jun 6;12(6):1019. doi: 10.3390/antibiotics12061019 (PMC10295116; doi:10.3390/antibiotics12061019)

Figure S3. Model predicted mean minimum inhibitory concentration (MIC) for enterococci/streptococci isolates from group-housed calves. Shaded areas represent 95% Confidence intervals.

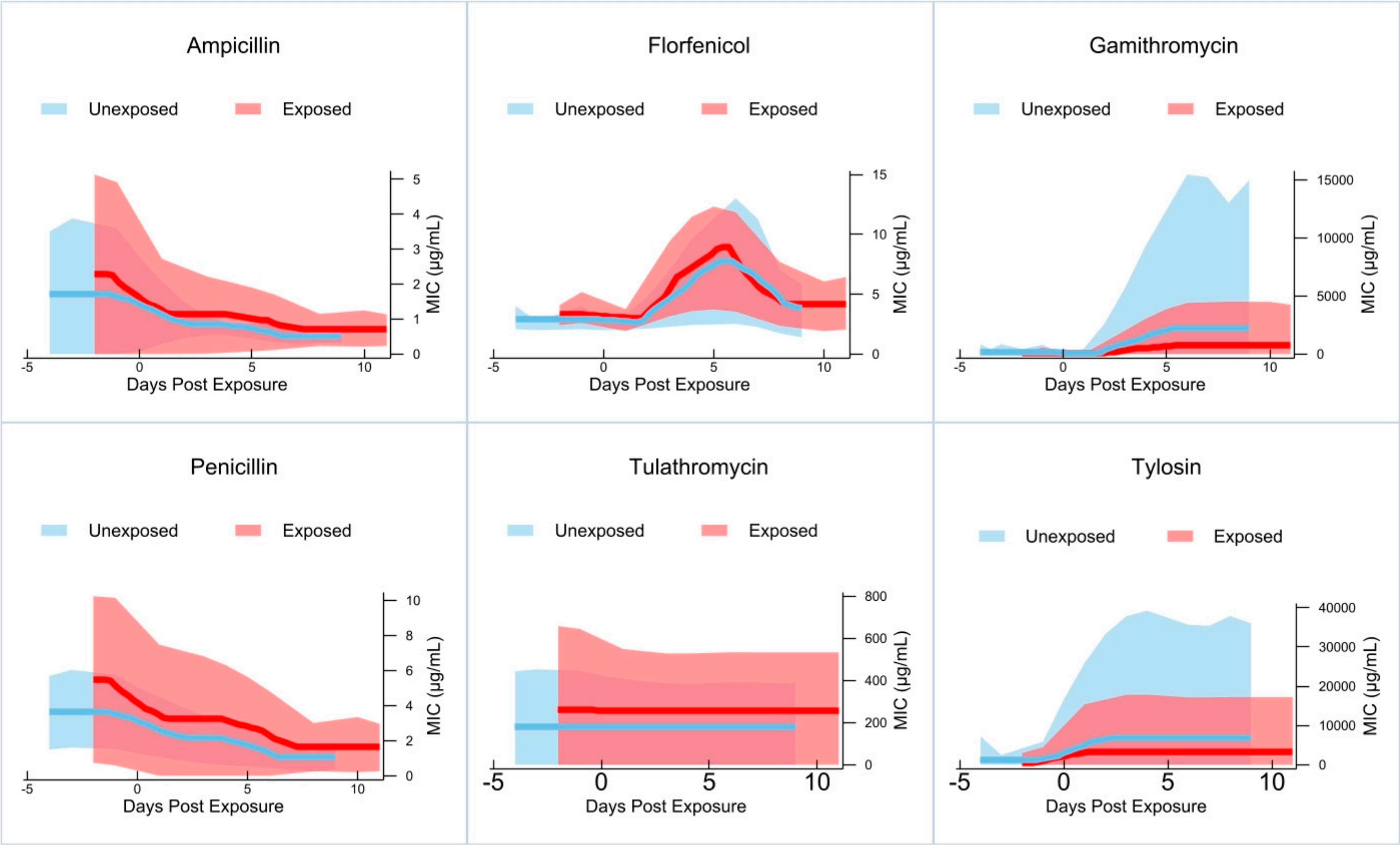

Supplement: Supplementary file 1 [file antibiotics-12-01019-s001.zip › Figure S3.pdf]
