# Supplementary material for: Effect of Group Housing of Preweaned Dairy Calves: Health and Fecal Commensal Antimicrobial Resistance Outcomes
Source: Antibiotics (Basel). 2023 Jun 6;12(6):1019. doi: 10.3390/antibiotics12061019 (PMC10295116; doi:10.3390/antibiotics12061019)
Supplement: Supplementary file 1 [file antibiotics-12-01019-s001.zip › Table S1.pdf]

Table S1a. *E. coli* minimum inhibitory concentration (MIC) Model results for Group-Housed Calves where enrofloxacin was the antimicrobial drug (AMD) of exposure. Coefficients represent MIC ratios for all AMD evaluated using Accelerated Failure Time Models for fixed effects of exposure, days post exposure (DPE), and their two-way interactions.

	Enrofloxacin		Ceftiofur		Danofloxacin	
Variable	MIC Ratio (95% CI)	p-value	MIC Ratio (95% CI)	p-value	MIC Ratio (95% CI)	p-value
Intercept	1.39 (0.522 3.692)	0.512	1.29 (0.358 4.676)	0.694	0.76 (0.308 1.875)	0.552
Unexposed	Referent		Referent		Referent	
Exposed	0.64 (0.140 2.907)	0.562	0.51 (0.039 6.692)	0.607	0.63 (0.152 2.593)	0.520
Before Exposure	Referent		Referent		Referent	
1-3 DPE	5.30 (1.074 26.110)	0.041	0.28 (0.077 1.044)	0.058	5.01 (1.069 23.476)	0.041
4-14 DPE	27.87 (3.045 255.071)	0.003	0.33 (0.073 1.494)	0.150	25.76 (2.847 233.191)	<0.01
Exposed x Before Exposure			Referent			
Exposed x 1-3 DPE			3.52 (0.207 59.805)	0.384		
Exposed x 4-14 DPE			50.86 (3.547 729.207)	<0.01		

Table S1b. *E. coli* minimum inhibitory concentration (MIC) Model results for Group-Housed Calves where enrofloxacin was the antimicrobial drug (AMD) of exposure. Coefficients represent MIC ratios for all AMD evaluated using Accelerated Failure Time Models for fixed effects of exposure, days post exposure (DPE), and their two-way interactions.

	Neomycin		Ampicillin	
Variable	MIC Ratio (95% CI)	p	MIC Ratio (95% CI)	p
Intercept	66.51 (23.021 192.165)	<0.01	8.49 (1.989 36.256)	<0.01
Unexposed	Referent		Referent	
Exposed	0.97 (0.142 6.637)	0.975	3.12 (0.322 30.319)	0.326
Before Exposure			Referent	
1-3 days			11.80 (2.481 56.169)	<0.01
4-14 DPE			34.48 (7.296 162.977)	<0.01
After exposure	6.13 (1.151 32.686)	0.034		

Table S1c. *E. coli* minimum inhibitory concentration (MIC) Model results for Group-Housed Calves where enrofloxacin was the antimicrobial drug (AMD) of exposure. Coefficients represent MIC ratios for all AMD evaluated using Accelerated Failure Time Models for fixed effects of exposure, days post exposure (DPE), and their two-way interactions.

	Tetracycline		Florfenicol		Spectinomycin	
Variable	MIC Ratio (95% CI)	p	MIC Ratio (95% CI)	p	MIC Ratio (95% CI)	p
Intercept	22.75 (2.491 207.761)	0.006	5.16 (3.357 7.921)	0.000	23.94 (13.753 41.659)	0.000
Unexposed	Referent		Referent		Referent	
Exposed	1.87 (0.132 26.474)	0.644	1.03 (0.565 1.888)	0.916	1.15 (0.608 2.162)	0.674
Before Exposure			Referent		Referent	
1-3 days			1.00 (0.534 1.884)	0.992	1.38 (0.593 3.210)	0.455
4-14 DPT			0.82 (0.388 1.721)	0.596	1.73 (0.671 4.475)	0.256
After exposure	11.54 (1.278 104.182)	0.029				
