# Supplementary material for: Effect of Group Housing of Preweaned Dairy Calves: Health and Fecal Commensal Antimicrobial Resistance Outcomes
Source: Antibiotics (Basel). 2023 Jun 6;12(6):1019. doi: 10.3390/antibiotics12061019 (PMC10295116; doi:10.3390/antibiotics12061019)
Supplement: Supplementary file 1 [file antibiotics-12-01019-s001.zip › Table S2.pdf]

Table S2a. *E. coli* Minimum Inhibitory Concentration (MIC) Model Predictions for Individual-Housed Calves where enrofloxacin was the antimicrobial drug (AMD) of exposure. Coefficients represent MIC ratios for all AMD evaluated using Accelerated Failure Time Models for fixed effects of exposure, days post exposure (DPE), and their two-way interactions.

	Enrofloxacin		Ceftiofur		Danofloxacin	
	Coefficient (95% CI)	p	Coefficient (95% CI)	p	Coefficient (95% CI)	p
Intercept	0.90 (0.134 6.086)	0.917	0.90 (0.303 2.651)	0.844	0.17 (0.055 0.528)	<0.01
Unexposed	Referent		Referent		Referent	
Exposed	0.87 (0.153 4.922)	0.874	2.58 (0.500 13.320)	0.258	5.18 (0.972 27.655)	0.054
Before Exposure	Referent		Referent		Referent	
1-3 days	4.70 (0.557 39.697)	0.155				
4-14 DPT	5.16 (1.314 20.253)	0.019				
1-3 DPT			2.42 (0.994 5.897)	0.052		
4-7 DPT			4.77 (0.448 50.814)	0.196		
8-14 DPT			47.41 (11.028 203.817)	<0.01		
1-5 DPT					64.70 (9.738 429.852)	<0.01
6-14 DPT					16.81 (0.700 403.822)	0.082
Exposed x Before Exposure			Referent		Referent	
Exposed x 1-3 DPT			0.25 (0.034 1.819)	0.170		
Exposed x 4-7 DPT			0.87 (0.026 29.485)	0.940		
Exposed x 8-14 DPT			0.01 (0.001 0.186)	<0.01		
Exposed x 1-5 DPT					0.03 (0.003 0.219)	<0.01
Exposed x 6-14 DPT					0.09 (0.003 2.475)	0.152

Table S2b. *E. coli* Minimum Inhibitory Concentration (MIC) Model Predictions for Individual-Housed Calves where enrofloxacin was the antimicrobial drug (AMD) of exposure. Coefficients represent MIC ratios for all AMD evaluated using Accelerated Failure Time Models for fixed effects of exposure, days post exposure (DPE), and their two-way interactions.

	Neomycin		Ampicillin		Gentamicin	
	Coefficient (95% CI)	p	Coefficient (95% CI)	p	Coefficient (95% CI)	p
Intercept	77.65 (20.797 289.948)	<0.01	12.05 (3.437 42.232)	<0.01	0.56 (0.228 1.368)	0.202
Unexposed	Referent		Referent		Referent	
Exposed	0.74 (0.244 2.245)	0.596	1.62 (0.506 5.182)	0.416	39.11 (10.173 150.381)	<0.01
Before Exposure	Referent		Referent		Referent	
1-3 days					87.79 (10.038 767.834)	<0.01
4-14 DPT					77.94 (12.913 470.420)	<0.01
1-14 DPT	4.30 (0.647 28.533)	0.131	9.07 (1.996 41.247)	<0.01		
Exposed x Before Exposure					Referent	
Exposed x 1-5 DPT					0.01 (0.001 0.169)	<0.01
Exposed x 6-14 DPT					0.17 (0.007 4.108)	0.276
Exposed x 1-3 DPT					0.01 (0.001 0.169)	<0.01
Exposed x 4-14 DPT					0.17 (0.007 4.108)	0.276

Table S2c. *E. coli* Minimum Inhibitory Concentration (MIC) Model Predictions for Individual-Housed Calves where enrofloxacin was the antimicrobial drug (AMD) of exposure. Coefficients represent MIC ratios for all AMD evaluated using Accelerated Failure Time Models for fixed effects of exposure, days post exposure (DPE), and their two-way interactions.

	Tetracycline		Florfenicol		Spectinomycin	
	Coefficient (95% CI)	p	Coefficient (95% CI)	p	Coefficient (95% CI)	p
Intercept	20.10 (5.529 73.099)	<0.01	5.28 (1.888 14.747)	<0.01	21.36 (5.799 78.707)	<0.01
Unexposed	Referent		Referent		Referent	
Exposed	0.76 (0.262 2.198)	0.611	4.36 (1.053 18.025)	0.042	1.24 (0.427 3.587)	0.694
Before Exposure	Referent		Referent		Referent	
1-3 DPT					4.00 (0.709 22.520)	0.116
4-7 DPT					2.45 (0.898 6.673)	0.080
8-14 DPT					2.77 (0.837 9.189)	0.095
1-5 DPT			8.43 (1.139 62.386)	0.037		
6-14 DPT			4.07 (1.121 14.800)	0.033		
1-14 DPT	4.13 (0.671 25.455)	0.126			0.45 (0.137 0.763)	<0.01
Exposed x Before Exposure			Referent		Referent	
Exposed x 1-5 DPT			0.06 (0.004 0.726)	0.027		
Exposed x 6-14 DPT			0.54 (0.059 4.878)	0.580		
