# Supplementary material for: Effect of Group Housing of Preweaned Dairy Calves: Health and Fecal Commensal Antimicrobial Resistance Outcomes
Source: Antibiotics (Basel). 2023 Jun 6;12(6):1019. doi: 10.3390/antibiotics12061019 (PMC10295116; doi:10.3390/antibiotics12061019)
Supplement: Supplementary file 1 [file antibiotics-12-01019-s001.zip › Table S3.pdf]

Table S3a. Results from Accelerated Failure Time Models for enterococci/streptococci isolates from Group-Housed calves exposed to an enrofloxacin treated calf. Coefficients represent minimum inhibitory concentration (MIC) ratios for all antimicrobial drugs evaluated using Accelerated Failure Time Models for fixed effects of exposure, days post exposure (DPE), and their two-way interactions.

	Ampicillin		Florfenicol		Gamithromycin	
	Coefficient (95% CI)	p	Coefficient (95% CI)	p	Coefficient (95% CI)	p
Intercept	1.70 (0.569 5.094)	0.341	2.94 (2.180 3.968)	<0.01	120.52 (3.481 4173.173)	<0.01
Unexposed	Referent		Referent		Referent	
Exposed	1.34 (0.569 3.142)	0.505	1.15 (0.765 1.718)	0.508	0.33 (0.026 4.245)	0.397
Before Exposure	Referent		Referent		Referent	
1-5 DPE	0.51 (0.152 1.681)	0.266				
6-14 DPE	0.31 (0.098 0.960)	0.042				
1-3 DPE			0.90 (0.652 1.242)	0.521	0.85 (0.044 16.477)	0.917
4-7 DPE			2.65 (1.394 5.041)	<0.01		
8-14 DPE			1.23 (0.728 2.086)	0.436		
4-14 DPE					18.19 (0.272 1216.853)	0.176

Table S3b. Results from Accelerated Failure Time Models for enterococci/streptococci isolates from Group-Housed calves exposed to an enrofloxacin treated calf. Coefficients represent minimum inhibitory concentration (MIC) ratios for all antimicrobial drugs evaluated using Accelerated Failure Time Models for fixed effects of exposure, days post exposure (DPE), and their two-way interactions.

	Tulathromycin		Tylosin		Penicillin	
	Coefficient (95% CI)	p	Coefficient (95% CI)	p	Coefficient (95% CI)	p
Intercept	261.91 (61.421 1116.849)	<0.01	1138.18 (10.465 1.2e+05)	<0.01	5.85 (2.852 11.994)	<0.01
Unexposed	Referent		Referent		Referent	
Exposed	1.43 (0.311 6.571)	0.646	0.49 (0.022 11.278)	0.659	1.51 (0.614 3.701)	0.370
Before Exposure	Referent		Referent		Referent	
1-5 DPE					0.60 (0.227 1.578)	0.299
6-14 DPE					0.31 (0.127 0.737)	<0.01
1-14 DPE	0.99 (0.260 3.779)	0.991	5.95 (0.179 197.803)	0.318		
