# Supplementary material for: Effect of Group Housing of Preweaned Dairy Calves: Health and Fecal Commensal Antimicrobial Resistance Outcomes
Source: Antibiotics (Basel). 2023 Jun 6;12(6):1019. doi: 10.3390/antibiotics12061019 (PMC10295116; doi:10.3390/antibiotics12061019)
Supplement: Supplementary file 1 [file antibiotics-12-01019-s001.zip › Table S4.pdf]

Table S4a. Results from Accelerated Failure Time Models for enterococci/streptococci isolates in Individual-Housed calves exposed to an enrofloxacin treated calf. Coefficients represent minimum inhibitory concentration (MIC) ratios for all antimicrobial drugs evaluated using Accelerated Failure Time Models for fixed effects of exposure, days post exposure (DPE), and their two-way interactions. Models for gamithromycin, tulathromycin and tylosin did not converge and are excluded.

	Ampicillin		Florfenicol	
	MIC Ratio (95% CI)	p	MIC Ratio (95% CI)	p
Intercept	0.42 (0.070 2.550)	0.347	2.93 (1.645 5.208)	<0.01
Unexposed	Referent		Referent	
Exposed	1.45 (0.294 7.125)	0.650	1.10 (0.541 2.218)	0.799
Before Exposure	Referent		Referent	
1-5 DPT	0.69 (0.213 2.260)	0.544		
6-14 DPT	1.52 (0.315 7.377)	0.601		
1-3 DPT			1.96 (0.933 4.096)	0.076
4-14 DPT			1.42 (0.738 2.717)	0.295

Table S4b. Results from Accelerated Failure Time Models for enterococci/streptococci isolates in Individual-Housed calves exposed to an enrofloxacin treated calf. Coefficients represent minimum inhibitory concentration (MIC) ratios for all antimicrobial drugs evaluated using Accelerated Failure Time Models for fixed effects of exposure, days post exposure (DPE), and their two-way interactions. Models for gamithromycin, tulathromycin and tylosin did not converge and are excluded.

	Penicillin		Tilmicosin	
	MIC Ratio (95% CI)	p	MIC Ratio (95% CI)	p
Intercept	3.25 (0.709 14.863)	0.129	18.36 (4.525 74.480)	<0.01
Unexposed	Referent		Referent	
Exposed	1.01 (0.171 5.949)	0.992	1.13 (0.372 3.401)	0.835
Before Exposure	Referent		Referent	
1-5 DPT	0.38 (0.084 1.725)	0.211		
6-14 DPT	0.99 (0.182 5.329)	0.987		
After exposure			3.21 (0.924 11.117)	0.066
