# Supplementary material for: Effect of Group Housing of Preweaned Dairy Calves: Health and Fecal Commensal Antimicrobial Resistance Outcomes
Source: Antibiotics (Basel). 2023 Jun 6;12(6):1019. doi: 10.3390/antibiotics12061019 (PMC10295116; doi:10.3390/antibiotics12061019)
Supplement: Supplementary file 1 [file antibiotics-12-01019-s001.zip › Table S5.pdf]

Table S5. Parameters for Accelerated Failure Time Models for *E. coli* and enterococci/streptococci isolates in Group-Housed and Individual-Housed calves exposed to an enrofloxacin treated calf.

Organism	Housing	Antimicrobial drug tested	Distribution	Number of observations	Number of right-censored observations	Number of left-censored observations	Model DF
EC	Group	Enrofloxacin	Exponential	66	49	14	3
		Ceftiofur	Lognormal	66	18	8	5
		Danofloxacin	Exponential	66	49	14	3
		Neomycin	Exponential	66	58	8	2
		Ampicillin	Lognormal	66	53	0	3
		Gentamicin	Lognormal	66	52	11	3
		Tetracycline	Lognormal	66	57	0	2
		Florfenicol	Lognormal	66	25	0	3
		Spectinomycin	Lognormal	66	24	3	3
	Individual	Enrofloxacin	Exponential	57	33	15	3
		Ceftiofur	Lognormal	57	19	1	7
		Danofloxacin	Exponential	57	33	16	5
		Neomycin	Exponential	57	49	8	2
		Ampicillin	Exponential	57	47	0	2
		Gentamicin	Exponential	57	40	13	5
		Tetracycline	Exponential	57	49	0	2
		Florfenicol	Exponential	57	39	0	5
		Spectinomycin	Lognormal	57	28	5	4
ES	Group	Ampicillin	Loglogistic	65	10	7	3
		Florfenicol	Loglogistic	65	19	1	4
		Gamithromycin	Lognormal	65	55	3	3
		Tulathromycin	Exponential	65	53	6	2
		Tylosin	Lognormal	65	55	1	2
		Penicillin	Weibull	65	13	7	3
	Individual	Ampicillin	Loglogistic	62	6	17	3
		Florfenicol	Lognormal	62	20	0	3
		Penicillin	Weibull	62	9	13	3
		Tilmicosin	Lognormal	62	49	0	2

Footnote: EC= *E. coli*, ES= enterococci/streptococci, DF= Degrees of Freedom
