# Supplementary material for: Effect of Group Housing of Preweaned Dairy Calves: Health and Fecal Commensal Antimicrobial Resistance Outcomes
Source: Antibiotics (Basel). 2023 Jun 6;12(6):1019. doi: 10.3390/antibiotics12061019 (PMC10295116; doi:10.3390/antibiotics12061019)
Supplement: Supplementary file 1 [file antibiotics-12-01019-s001.zip › Table S6.pdf]

Table S6. Predicted minimum inhibitory concentration (MIC) to all drugs evaluated using Accelerated Failure Time models for exposed, unexposed and the estimated difference between them (exposed-unexposed) for *E. coli* from individual-housed calves where enrofloxacin was the antimicrobial drug (AMD) of exposure. Minimum inhibitory concentration (MIC) predictions were calculated using the exponentiated coefficients from the model results.

		Exposed		Unexposed		Exposed-Unexposed	
Drug	Variable Level	MIC (95% CI)	p	MIC (95% CI)	p	MIC Difference (95% CI)	p
Enrofloxacin	Pre-Exposure	0.79 (-0.098 1.669)	0.081	0.90 (-0.820 2.627)	0.304	-0.12 (-1.669 1.433)	0.881
	1-3 DPE	3.69 (-3.341 10.724)	0.304	4.25 (-0.329 8.824)	0.069	-0.56 (-7.070 5.959)	0.867
	4-11 DPE	4.05 (-1.172 9.275)	0.128	4.66 (-1.658 10.981)	0.148	-0.61 (-8.220 7.000)	0.875
Ceftiofur	Pre-Exposure	2.31 (-0.598 5.225)	0.119	0.90 (-0.075 1.869)	0.071	1.42 (-1.631 4.465)	0.362
	1-3 DPE	1.39 (-0.227 3.006)	0.092	2.17 (0.585 3.759)	0.007	-0.78 (-3.012 1.447)	0.492
	4-7 DPE	9.65 (-5.062 24.359)	0.199	4.28 (-6.892 15.449)	0.453	5.37 (-12.870 23.611)	0.564
	8-11 DPE	1.27 (-0.749 3.285)	0.218	42.52 (-33.863 118.910)	0.275	-41.26 (-117.594 35.083)	0.289
Danofloxacin	Pre-Exposure	0.89 (-0.21, 1.98)	0.11	0.17 (-0.02, 0.36)	0.08	0.72 (-0.39, 1.83)	0.21
	1-5 DPE	1.48 (-0.16, 3.13)	0.07	11.06 (-9.70, 31.82)	0.29	-9.58 (-30.40, 11.25)	0.37
	6-11 DPE	0.39 (-0.79, 1.57)	0.52	2.87 (-3.47, 9.21)	0.37	-1.60 (-8.02, 4.82)	0.66
Neomycin	Pre-Exposure	57.50 (-37.097 152.105)	0.234	77.65 (-24.651 179.957)	0.137	-20.15 (-91.385 51.088)	0.579
	Post-Exposure	247.10 (20.438 473.756)	0.033	333.68 (-13.594 680.945)	0.060	-86.58 (-425.320 252.164)	0.616
Ampicillin	Pre-Exposure	19.51 (-3.627 42.650)	0.098	12.05 (-3.064 27.159)	0.118	7.46 (-12.062 26.990)	0.454
	Post-Exposure	177.03 (-14.537 368.588)	0.070	109.31 (10.157 208.455)	0.031	67.72 (-116.992 252.430)	0.472
Gentamicin	Pre-Exposure	21.83 (-0.101 43.760)	0.051	0.56 (0.058 1.059)	0.029	21.27 (-0.665 43.208)	0.057
	1-3 DPE	21.83 (-15.085 58.744)	0.246	49.00 (-62.541 160.538)	0.389	-27.17 (-144.658 90.320)	0.650
	4-11 DPE	289.50 (-260.856 839.856)	0.303	43.50 (-34.661 121.657)	0.275	246.00 (-309.877 801.880)	0.386
Tetracycline	Pre-Exposure	15.26 (-8.256 38.769)	0.203	20.10 (-5.848 46.057)	0.129	-4.85 (-23.115 13.419)	0.603
	Post-Exposure	63.06 (5.656 120.464)	0.031	83.10 (0.140 166.055)	0.050	-20.04 (-100.904 60.830)	0.627
Spectinomycin	Pre-Exposure	26.45 (0.093 52.801)	0.049	21.36 (-6.495 49.223)	0.133	5.08 (-19.169 29.337)	0.681
	1-3 DPE	105.67 (-60.768 272.111)	0.213	85.36 (-44.161 214.880)	0.196	20.31 (-87.114 127.738)	0.711
	4-7 DPE	64.76 (7.321 122.194)	0.027	52.31 (-0.505 105.124)	0.052	12.45 (-49.226 74.122)	0.692
	8-11 DPE	73.33 (20.612 126.044)	0.006	59.23 (-20.539 139.005)	0.146	21.02 (-56.647 98.684)	0.596
Florfenicol	Pre-Exposure	22.99 (0.453 45.518)	0.046	5.28 (-0.146 10.700)	0.056	17.71 (-5.467 40.884)	0.134
	1-5 DPE	10.97 (-0.024 21.963)	0.051	44.48 (-9.109 98.071)	0.104	-33.51 (-88.218 21.194)	0.230
	6-11 DPE	50.25 (-9.982 110.474)	0.102	21.50 (0.529 42.463)	0.044	28.75 (-35.024 92.523)	0.377
