# Supplementary material for: Effect of Group Housing of Preweaned Dairy Calves: Health and Fecal Commensal Antimicrobial Resistance Outcomes
Source: Antibiotics (Basel). 2023 Jun 6;12(6):1019. doi: 10.3390/antibiotics12061019 (PMC10295116; doi:10.3390/antibiotics12061019)
Supplement: Supplementary file 1 [file antibiotics-12-01019-s001.zip › Table S7.pdf]

Table S7. Predicted minimum inhibitory concentration (MIC) to all drugs evaluated using Accelerated Failure Time models for exposed, unexposed and the estimated difference between them (exposed-unexposed) for *E. coli* from Group-housed calves where enrofloxacin was the antimicrobial drug (AMD) of exposure. Mean MIC predictions were calculated using the exponentiated coefficients from the model results.

		Exposed		Unexposed		Exposed - Unexposed	
Drug	Variable Level	MIC (95% CI)	p	MIC (95% CI)	p	MIC Difference (95% CI)	p
Enrofloxacin	Pre-Exposure	0.89 (-0.514 2.286)	0.215	1.39 (0.030 2.746)	0.045	-0.50 (-2.045 1.042)	0.524
	1-3 DPE	4.69 (-1.247 10.635)	0.122	7.35 (-1.724 16.422)	0.112	-2.66 (-12.089 6.778)	0.581
	4-11 DPE	24.70 (-10.414 59.819)	0.168	38.68 (-50.067 127.421)	0.393	-13.97 (-79.870 51.920)	0.678
Ceftiofur	Pre-Exposure	0.66 (-0.820 2.135)	0.383	1.29 (-0.368 2.957)	0.127	-0.64 (-2.851 1.578)	0.573
	1-3 DPE	0.66 (-0.293 1.608)	0.175	0.37 (0.259 0.477)	0.000	0.29 (-0.665 1.245)	0.552
	4-11 DPE	11.05 (1.597 20.494)	0.022	0.43 (0.136 0.719)	0.004	10.62 (1.171 20.066)	0.028
Danofloxacin	Pre-Exposure	0.48 (-0.240 1.195)	0.192	0.76 (0.074 1.447)	0.030	-0.28 (-1.058 0.492)	0.475
	1-3 DPE	2.39 (-0.576 5.362)	0.114	3.81 (-0.620 8.239)	0.092	-1.42 (-5.934 3.101)	0.539
	4-11 DPE	12.31 (-5.243 29.856)	0.169	19.59 (-24.396 63.579)	0.383	-7.29 (-39.253 24.683)	0.655
Neomycin	Pre-Exposure	64.49 (-74.699 203.682)	0.364	66.51 (-4.055 137.079)	0.065	-2.02 (-126.176 122.135)	0.975
	Post-Exposure	395.63 (-144.177 935.443)	0.151	408.03 (-162.580 978.634)	0.161	-12.39 (-785.712 760.924)	0.975
Ampicillin	Pre-Exposure	26.52 (-34.081 87.126)	0.391	14.07 (1.501 26.639)	0.028	18.03 (-39.744 75.804)	0.541
	1-3 DPE	313.09 (-462.062 1088.234)	0.429	75.46 (-17.869 168.787)	0.113	212.84 (-495.329 921.003)	0.556
	4-11 DPE	914.60 (-1.1e+03 2882.663)	0.362	170.41 (-181.114 521.938)	0.342	621.75 (-1.1e+03 2307.688)	0.470
Tetracycline	Pre-Exposure	42.51 (-92.488 177.517)	0.537	22.75 (-27.569 73.071)	0.376	19.76 (-94.56, 134.089)	0.735
	Post-Exposure	490.50 (-634.407 1615.414)	0.393	262.48 (-382.531 907.493)	0.425	228.02 (-835.742, 1291.79)	0.674
Florfenicol	Pre-Exposure	5.33 (1.764 8.892)	0.003	5.16 (2.944 7.370)	0.000	0.17 (-3.027 3.369)	0.917
	1-3 DPE	5.34 (1.511 9.179)	0.006	5.17 (1.806 8.541)	0.003	0.17 (-3.013 3.356)	0.916
	4-14 DPE	4.35 (1.836 6.873)	0.001	4.21 (1.799 6.631)	0.001	0.14 (-2.445 2.724)	0.916
Spectinomycin	Pre-Exposure	27.43 (7.105 47.755)	0.008	23.94 (10.672 37.200)	0.000	3.49 (-13.573 20.562)	0.688
	1-3 DPE	37.85 (13.063 62.634)	0.003	33.03 (12.203 53.851)	0.002	4.82 (-17.944 27.587)	0.678
	4-14 DPE	47.54 (8.262 86.809)	0.018	41.48 (16.432 66.528)	0.001	6.06 (-23.925 36.036)	0.692
