# Supplementary material for: Effect of Group Housing of Preweaned Dairy Calves: Health and Fecal Commensal Antimicrobial Resistance Outcomes
Source: Antibiotics (Basel). 2023 Jun 6;12(6):1019. doi: 10.3390/antibiotics12061019 (PMC10295116; doi:10.3390/antibiotics12061019)
Supplement: Supplementary file 1 [file antibiotics-12-01019-s001.zip › Table S8.pdf]

Table S8. Predicted minimum inhibitory concentration (MIC) to all drugs evaluated using Accelerated Failure Time models for exposed, unexposed and the estimated difference between them (exposed-unexposed) for enterococi/streptococci from Individual-housed calves where enrofloxacin was the antimicrobial drug (AMD) of exposure. Minimum inhibitory concentration (MIC) predictions were calculated using the exponentiated coefficients from the model results.

		Exposed		Unexposed		Exposed - Unexposed	
Drug	Variable Level	MIC (95% CI)	p	MIC (95% CI)	p	MIC difference (95% CI)	p
Ampicillin	Pre-Exposure	0.59 (-0.274 1.449)	0.181	0.51 (-0.490 1.517)	0.316	0.07 (-0.892 1.040)	0.881
	1-5 DRT	0.37 (0.132 0.604)	<0.01	0.32 (-0.229 0.872)	0.252	0.05 (-0.545 0.638)	0.878
	5-14 DRT	1.06 (0.242 1.884)	0.011	0.93 (-0.969 2.827)	0.337	0.13 (-1.537 1.805)	0.875
Florfenicol	Pre-Exposure	3.18 (1.200 5.170)	<0.01	2.96 (1.208 4.717)	<0.01	0.22 (-1.995 2.440)	0.844
	Post-Exposure	4.95 (1.759 8.145)	<0.01	4.61 (2.167 7.045)	<0.01	0.35 (-3.125 3.817)	0.845
Penicillin	Pre-Exposure	3.28 (-1.301 7.854)	0.161	3.25 (-1.693 8.185)	0.198	0.03 (-5.752 5.812)	0.992
	1-5 DRT	1.25 (0.321 2.177)	<0.01	1.24 (-1.123 3.597)	0.304	0.01 (-2.183 2.206)	0.992
	5-14 DRT	3.23 (0.456 6.005)	0.023	3.20 (-2.950 9.350)	0.308	0.03 (-5.648 5.708)	0.992
Tilmicosin	Pre-Exposure	20.66 (-11.224 52.536)	0.204	18.36 (-7.351 44.069)	0.162	2.30 (-19.938 24.533)	0.840
	Post-Exposure	66.21 (18.252 114.178)	<0.01	58.85 (-6.177 123.878)	0.076	7.36 (-59.685 74.414)	0.830
