# Supplementary material for: Effect of Group Housing of Preweaned Dairy Calves: Health and Fecal Commensal Antimicrobial Resistance Outcomes
Source: Antibiotics (Basel). 2023 Jun 6;12(6):1019. doi: 10.3390/antibiotics12061019 (PMC10295116; doi:10.3390/antibiotics12061019)
Supplement: Supplementary file 1 [file antibiotics-12-01019-s001.zip › Table S9.pdf]

Table S9. Predicted minimum inhibitory concentration (MIC) to all drugs evaluated using Accelerated Failure Time models for exposed, unexposed and the estimated difference between them (exposed-unexposed) for enterococi/streptococci from Group-housed calves where enrofloxacin was the antimicrobial drug (AMD) of exposure. Minimum inhibitory concentration (MIC) predictions were calculated using the exponentiated coefficients from the model results.

		Exposed		Unexposed		Exposed - Unexposed	
Drug	Variable Level	MIC (95% CI)	p	MIC (95% CI)	p	MIC Difference (95% CI)	p
Ampicillin	Pre-Exposure	3.20 (-0.441 6.840)	0.085	2.21 (0.088 4.333)	0.041	0.99 (-1.920 3.898)	0.505
	1-3 DPE	1.32 (0.051 2.595)	0.041	0.91 (0.068 1.760)	0.034	0.41 (-0.718 1.536)	0.477
	4-7 DPE	1.18 (-0.140 2.507)	0.080	0.82 (0.540 1.095)	<0.01	0.37 (-0.809 1.541)	0.541
	8-14 DPE	0.76 (-0.017 1.539)	0.055	0.53 (0.122 0.930)	0.011	0.24 (-0.454 0.924)	0.503
Florfenicol	Pre-Exposure	2.83 (2.828 2.828)	<0.01	3.54 (2.184 4.891)	<0.01	-0.71 (-2.063 0.645)	0.305
	1-5 DPE	3.48 (1.353 5.614)	<0.01	4.76 (3.142 6.381)	<0.01	-1.28 (-4.075 1.520)	0.371
	6-14 DPE	6.24 (2.805 9.665)	<0.01	3.62 (1.247 5.994)	<0.01	2.61 (-1.313 6.542)	0.192
Gamithromycin	Pre-Exposure	40.04 (-98.629 178.706)	0.571	120.52 (-306.685 547.734)	0.580	-80.49 (-418.939 257.966)	0.641
	1-3 DPE	34.21 (-61.303 129.730)	0.483	102.99 (-223.959 429.941)	0.537	-68.78 (-344.312 206.757)	0.625
	4-14 DPE	728.41 (-2.9e+03 4335.421)	0.692	2192.68 (-1.0e+04 1.4e+04)	0.726	-1464.27 (-1.1e+04 7727.313)	0.755
Tulathromycin	Pre-Exposure	374.48 (-196.692 945.646)	0.199	261.91 (-117.928 641.751)	0.177	112.57 (-401.420 626.552)	0.668
	Post exposure	371.46 (-26.095 769.020)	0.067	259.80 (-42.937 562.542)	0.093	111.66 (-370.613 593.932)	0.650
Tylosin	Pre-Exposure	613.64 (-1.8e+03 2978.694)	0.611	747.06 (-2.3e+03 3816.749)	0.633	-133.42 (-2.3e+03 2058.796)	0.905
	Post exposure	3928.05 (-1.1e+04 1.9e+04)	0.597	4782.09 (-1.5e+04 2.4e+04)	0.632	-854.04 (-1.5e+04 1.3e+04)	0.906
Penicillin	Pre-Exposure	8.28 (1.919 14.634)	0.011	5.85 (1.648 10.049)	<0.01	2.97 (-4.521 10.463)	0.437
	1-5 DPE	5.30 (0.758 9.848)	0.022	3.50 (1.599 5.398)	<0.01	1.78 (-3.013 6.567)	0.467
	6-14 DPE	2.87 (0.716 5.017)	<0.01	1.79 (0.610 2.962)	<0.01	0.91 (-1.288 3.103)	0.418

*DPE=Days Post Exposure
